# Hydrophobilization of Furan-Containing Polyurethanes via Diels–Alder Reaction with Fatty Maleimides

**DOI:** 10.3390/polym11081274

**Published:** 2019-07-31

**Authors:** Philipp Schmidt, Steven Eschig

**Affiliations:** Fraunhofer Institute for Wood Research, Wilhelm-Klauditz-Institut WKI, 38108 Braunschweig, Germany

**Keywords:** post-polymerization modification, polyurethanes, Diels–Alder addition, hydrophobicity, customized polymers, furan, maleimide, surface modification

## Abstract

We describe new hydrophobic functionalized linear polyurethane resins by combining N-alkyl maleimides via the Diels–Alder reaction with linear furan-modified polyurethanes. This procedure provides the opportunity for the post-polymerization-functionalizing of polyurethanes. Access to furan-bearing polyurethanes is achieved via the reaction of a furan-containing diol, polyethylenglycol (PEG), and different diisocyanates. The furan-containing diol is obtained from the reaction of furfurylamine and two equivalents of hydroxyalkyl acrylate. The resulting furan-bearing polyurethanes are reacted with fatty amine-based N-alkyl maleimides. The maleimide and furan functionalities undergo a Diels–Alder reaction, which allows for the covalent bonding of the hydrophobic side chains to the polyurethane backbone. The covalent bonding of the hydrophobic maleimides to the polyurethane backbone is proven by means of NMR. The influence of the functionalization on the surface properties of the resulting polyurethane films is analyzed via the determination of surface energy via the sessile drop method.

## 1. Introduction

Polyurethanes have a high variety of applications including coatings [1], foams [2,3], fibers [4,5,6], adhesives [7], and thermoplastic elastomers [5,8,9]. They are obtained from the reaction of isocyanates and alcohols. Through the utilization of hard and soft segments, polyurethanes can be designed to have various unique properties [6].

Much attention is also paid to the degradability and recycling of such polymers [10]. Another field of research is the functionalization of polyurethane materials either during step-growth polymerization or afterwards [11,12,13]. The application of polyurethanes in advanced technology requires highly functionalized polyurethanes, e.g., hydrophobic modifications [1,14], self-healing abilities [15], or hydrophilic properties [16,17], to alter or custom fit surface properties.

In order to raise hydrophobic properties afterwards, low-surface-energy compounds need to be introduced [1,15]. A common approach in the preparation of hydrophobic polyurethanes is to utilize hydrophobic monomers or particles [15,18]. Recently, investigations have focused on polymer nanocomposites made by introducing inorganic nanoparticles to polymers to increase hydrophobicity [15,19,20,21].

A promising post-polymerization method to modify polyurethanes and other polymers is the [4 + 2]-cycloaddition of maleimides and furans [13,15,22,23,24,25]. There are different synthetic approaches described in the literature to utilize maleimides and furan for polymeric products. Bozell et al. reviewed the top bio-based products from biorefinery carbohydrates. They report that 2,5-furandicarboxylic acid (FDCA), furfural, and hydroxymethylfurfural (HMF) were the utmost important furan-based starting materials [26]. Souza et al. used FDCA as a building block for furan-containing polyesters [27]. Vijjamarri et al. utilized bishydroxymethylfuran (BHMF), 2,5-diformylfuran (DFF) and 5,5′- [oxybis(methylene)]di(2-furaldehyde) (OBMF) to synthesize different polymeric silylethers [28]. Recently, Xu et al. investigated furfurylamine as a building block, which was reacted with glycidyl ether [29]. Several strategies were also developed to introduce furan and maleimide moieties into polymeric materials by Gandini et al. [22].

To our knowledge, the difunctional furans BHMF, OBMF, and DFF are not yet available at an industrial scale. Thus, these compounds are very cost-intensive [30]. For example, the costs for BHMF are 20,000 $/t. In contrast, the monofunctional furans furfuryl alcohol and furfurylamine are already accessible at an industrial scale [31]. The cost is 1600 $/t for the alcohol and 6000–8000 $/t for the amine. As both furan components contain only one functional group, they are only suitable for end-capping purposes [29,32].

Reversible Diels–Alder reactions attract more and more attention to the preparation of highly functionalized polymers [33,34,35,36,37]. In this study we describe a method to synthesize functional customizable polyurethanes bearing furan groups. These polyurethanes are functionalized by introducing N-alkyl maleimides via a [4 + 2]-cycloaddition to raise hydrophobic properties of the polyurethanes. The N-alkyl maleimides are synthesized via a recently published synthetic method developed by us [18].

## 2. Materials and Methods

### 2.1. Instruments

^1^H-NMR and ^13^C-NMR spectra were recorded on a Bruker (Billerica, MA, USA) AV III-400 (400.1 MHz for ^1^H, 100.6 MHz for ^13^C, *T* = 296 K), Bruker DRX-400 (400.1 MHz for ^1^H, 100.6 MHz for ^13^C, *T* = 300 K), and Bruker AV II-600 (600.1 MHz for ^1^H, 150.9 MHz for ^13^C, *T* = 295 K) using CDCl_3_ or THF-d_8_ as a solvent. Chemical shifts are expressed in ppm with TMS (0.00 ppm) as an internal standard for ^1^H. ^13^C-NMR spectra were referenced to CDCl_3_ (77.0 ppm) or THF-d_8_ (67.2 ppm and 15.3 ppm). IR spectra were measured with a Nicolet iS5 iD7 ATR (ThermoFisher Scientific, Waltham, MA, USA). Mass spectra (ESI-MS/EI-MS) were performed on Finnigan MAT 95 XL (Thermofinnigan MAT, Waltham, MA, USA) and LTQ Orbitrap Velos (ThermoFisher Scientific, Waltham, MA, USA) spectrometers. Viscosities were measured on a Bohlin CVO 100 Rheometer (Worcestershire, UK) at 90 °C with a constant shearing stress of 100 Pa. Contact angle measurements were achieved with the sessile drop method on DataPhysics Instruments SCA20 (Filderstadt, Germany). Glass transition temperature (T_g_) were measured on a Mettler Toledo DSC 3+ in the range of −70 to 180 °C under a nitrogen atmosphere. The heating rate was kept at 10 K/min. DSC data were processed by Mettler STARe software (version 15.a) and exploited by ASTM E1356 standards.

### 2.2. Chemicals

Solvents and reagents were obtained from Carl Roth (polyethylene glycol 400 (PEG 400)), Alfa Aesar (2-hydroxyethyl acrylate (HEA), furfurylamine, 1,1′-methylenebis(4-isocyanatobenzene) (MDI)), and VWR International (chloroform). 4-Hydroxybutyl acrylate was provided by BASF and 5-isocyanato-1-(isocyanatomethyl)-1,3,3-trimethylcyclohexane (IPDI) by Evonik Industries. 1,6-Diisocyanatohexane (HDI) was obtained by Perstop AB (Malmö, Sweden). Borchikat^®^ 0244 was provided by OMG Borchers GmbH (Langenfeld, Germany). Maleimide STD was achieved by reaction of maleic anhydride (Alfa Aesar) with a mixture of fatty amines (Rofamin STD) provided by Ecogreen Oleochemicals. All chemicals were of synthesis quality and were used without further purification.

### 2.3. General Procedure for Synthesis of Michael Products

A 2000 mL round bottom flask was charged with hydroxyalkyl acrylate (6.974 mol, 2 eq.) and cooled in an ice bath. A dropping funnel with pressure compensation was filled with furfurylamine (3.487 mol, 1 eq.) and fitted to the round bottom flask. Under constant ice bath cooling and vigorous stirring, furfurylamine was added dropwise over 1 h to the hydroxyalkyl acrylate. The solution was stirred for an additional 3 h while cooling in an ice bath. Afterwards, the reaction mixture was stirred for 6 days at room temperature (rt). Progress of the reaction was tracked by thin-layer chromatography (TLC). After 6 days the product was obtained as a yellow oil in quantitative yields. The product was used without further purification in the next step.

### 2.4. General Procedure for Synthesis of Linear Polyurethanes

A 100 mL three necked round bottom flask was charged with PEG 400, the furfuryl-containing Michael addition product, and the diisocyanates (HDI, MDI, IPDI). The sum of all initial weights was 20 g. The molar ratio of PEG 400 and Michael adducts was defined as 1:1. The NCO/OH value was set to 1:1. The molar diisocyanate ratios of the different batches are summarized in Table 1. The reaction mixture was solved in dry acetone (12 mL) and diluted to give a 60 wt% solution. The solution was heated up to 60 °C while stirring. Depending on the viscosity of the mixture, additional acetone was added. At 60 °C, 0.06 m% Borchikat^®^ 0244 was added to the mixture and stirred for 4 h. After 4 h the reaction progress was checked by IR. With the depletion of isocyanates the reaction was finished, giving the polyurethanes as yellow resins. The products were used without further purification.

### 2.5. General Procedure for Diels–Alder Reaction of Linear Polyurethanes

A 50 mL round bottom flask was charged with polyurethane (2–3 g) and 80 mol% of maleimide STD related to the quantity of furan groups in the polyurethane. The reactants were solved in 3–4 mL of chloroform and diluted to a 40 wt% solution. The solution was refluxed under constant stirring for 16 h. After the end of the reaction, a plain glass plate was coated with a 250 μm layer of the solution. Then the product was dried for 2–3 days at rt. After drying, the product appeared as a light yellow resin and its hydrophobic properties were further analyzed (sessile drop method).

## 3. Results and discussion

### 3.1. Synthesis of Furfurylated Products via Michael Addition

**Scheme 1:**
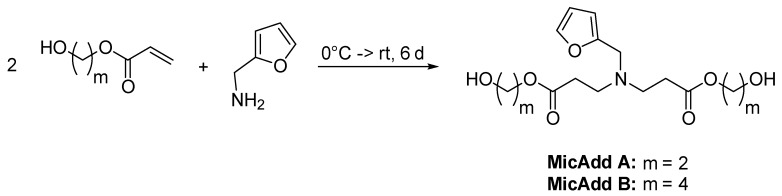
Michael addition of hydroxyalkyl acrylate and furfurylamine to access a tertiary amino ester.

The synthesis of linear furfurylated polyurethanes requires difunctional furan-containing building blocks e.g., bishydroxymethylfuran (BHMF) [30,38]. As BHMF is still too expensive, an alternative synthesis for a furan-containing diol was developed. The reaction of furfurylamine with hydroxyalkyl acrylates, following the procedure of Tseng et al., was an appropriate way to generate the required diol. They reported on the synthesis of the hydroxyfunctional tertiary amino ester [39]. They showed that the reaction of glycol acrylate and primary amines gives the required tertiary amino esters in almost quantitative yields via a Michael reaction. Therefore, we used the same procedure for the reaction of furfurylamine and hydroxyalkyl acrylate to obtain a furan-containing diol (Scheme 1).

**Figure 1:**
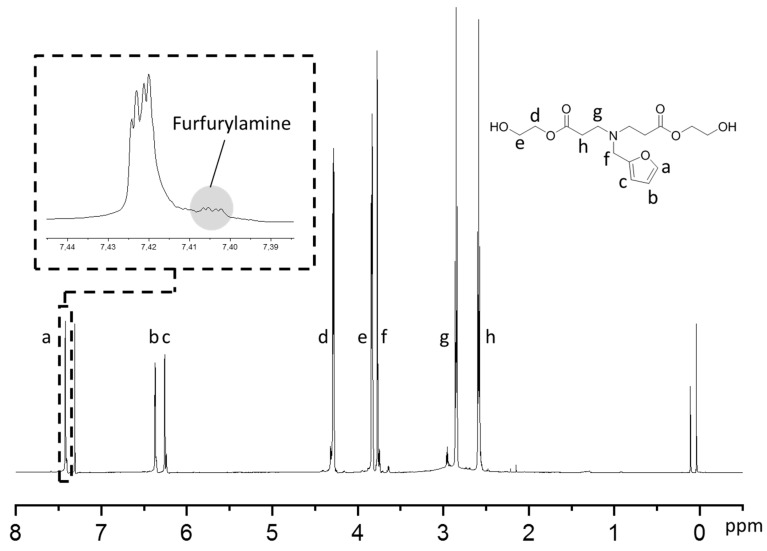
^1^H-NMR MicAdd B; product containing traces of furfurylamine.

The reaction was carried out for 6 days at rt. TLC showed only slight traces of non-converted furfurylamine. The spot for hydroxyalkyl acrylate had vanished. IR spectra showed the disappearance of the absorptions at 1638 cm^−1^ and 1618 cm^−1^, which are characteristic for the carbon double bond of hydroxyalkyl acrylate. Characteristic absorptions of the amino group in the range of 3100–3500 cm^−1^ were overlaid by absorptions of the hydroxyl groups. However, the absorption of the amine at 1601 cm^−1^ (scissoring) disappeared as well. Therefore, the reaction was considered to be finished. ^1^H-NMR analysis of the product mixture showed traces of 0.8 mol% of non-converted furfurylamine. The spectra are illustrated in Figure 1. The amount was determined by integrating the C1-proton signal of the furan group at 7.42 ppm (Michael addition product) and 7.40 ppm (furfurylamine). Thus, the overall yield of the reaction can be considered as almost quantitative. The furfurylated Michael adducts could be used without further purification for the next steps.

### 3.2. Synthesis of Linear Polyurethanes (PUs)

**Scheme 2:**
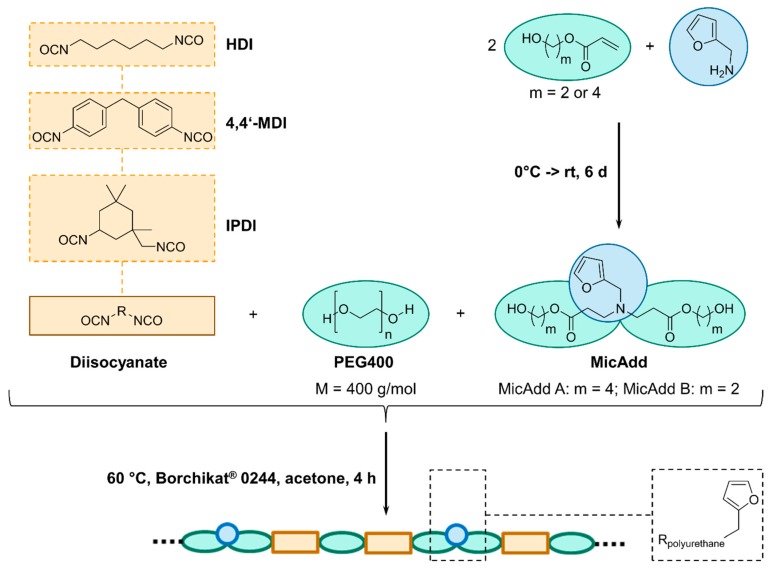
Process for synthesis of linear furan-containing polyurethanes.

Linear polyurethanes (PUs) were synthesized via the reaction of the furan-containing diols, Michael adduct A (MicAdd A) and Michael adduct B (MicAdd B), different isocyanates, and PEG 400. The process is shown in Scheme 2. Different combinations of diisocyanates HDI, IPDI, and MDI as well as Michael addition products were used to adapt physical properties, e.g., hardness, viscosity, and glass transition temperature (T_g_). An overview of the different batch compositions is given in Table 1. Reactions were carried out at 60 °C in a 40 wt% solution in acetone. As viscosities of reactions with high amounts of IPDI or MDI increased quickly, we had to add additional amounts of acetone to keep on stirring constantly. Reaction progress was tracked by IR. The characteristic absorptions of the isocyanate functionalities were detected at 2241 cm^−1^ for IPDI, 2249 cm^−1^ for HDI, and 2258 cm^−1^ for MDI. As the isocyanate groups react with the hydroxyl function of the used diols to form urethane groups, the isocyanate absorption decreased with reaction progress. The reaction was considered to be finished with a complete disappearance of those absorptions. ^1^H-NMR and ^13^C-NMR spectra showed the successful introduction of furan groups into the polyurethane backbones. ^1^H-NMR shifts were found at 7.42 ppm for H1, 6.38 ppm for H2, and 6.26 ppm for H3, which are characteristic for the furan ring. C-shifts of the furan ring were determined at 151 ppm for C1, 142 ppm for C4, 110 ppm for C2, and 108 ppm for C3 in the ^13^C-NMR spectrum.

Physical properties of the polyurethanes were measured to determine the influence of the monomers. Viscosities were measured at 90 °C with a constant shearing stress of 100 Pa.

For the polyurethanes containing 100 mol%, HDI viscosities of 8 Pas for PU01A and 78 Pas for PU01B were measured. This shows that longer alkyl chains in MicAdd A lead to a lower viscosity than the shorter chains in MicAdd B, as expected.

**Figure 2:**
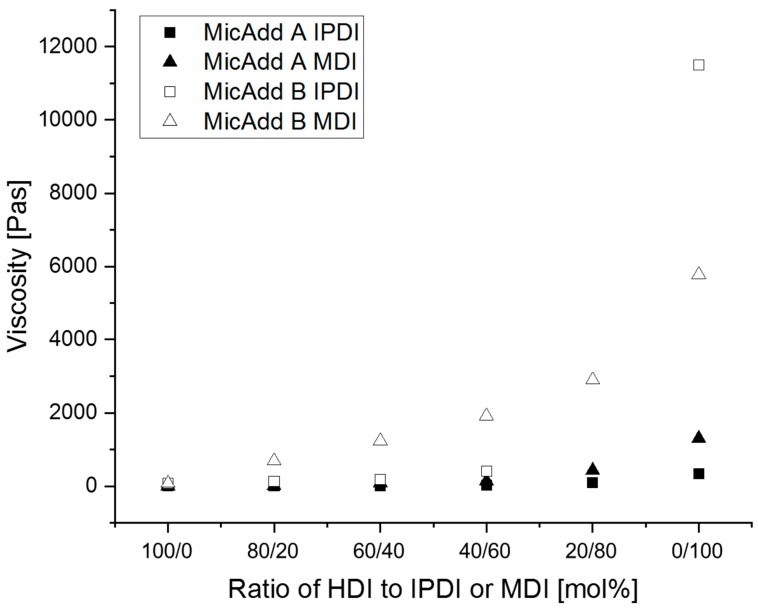
Change of viscosity by altering ratios of diisocyanates in the reaction mixture.

Raising the amount of the IPDI/MDI showed an overall increase of viscosity of the polyurethanes. This is illustrated in Figure 2. The polyurethanes containing MicAdd A and IPDI showed only a low increase of viscosity. For PU06A, which was synthesized with 100 mol% IPDI, a viscosity of 342 Pas was measured. The same tendencies were detected for polyurethanes PU07A to PU11A containing MDI. The viscosity of PU11A with 100% MDI was determined to be 436 Pas.

In comparison, the samples of PU01B–11B containing MicAdd B showed higher overall viscosities than polyurethanes PU01A–11A containing MicAdd A. The difference between those Michael products is the length of the carbon chain. MicAdd A contains a butyl moiety and MicAdd B an ethyl moiety. The longer carbon chain leads to an observable effect on viscosity.

Viscosities of polyurethanes PU05B and PU06B exceed the expected values. Moreover, it was not possible to measure the viscosity for PU05B at a temperature of 90 °C. Viscosities were expected to increase exponentially with an ascending amount of IPDI, as shown in Figure 2, but to be lower overall than their corresponding MDI series. Repetition of these experiments under the same conditions gave the same results. So far, these unexpected deviations cannot be explained.

**Figure 3:**
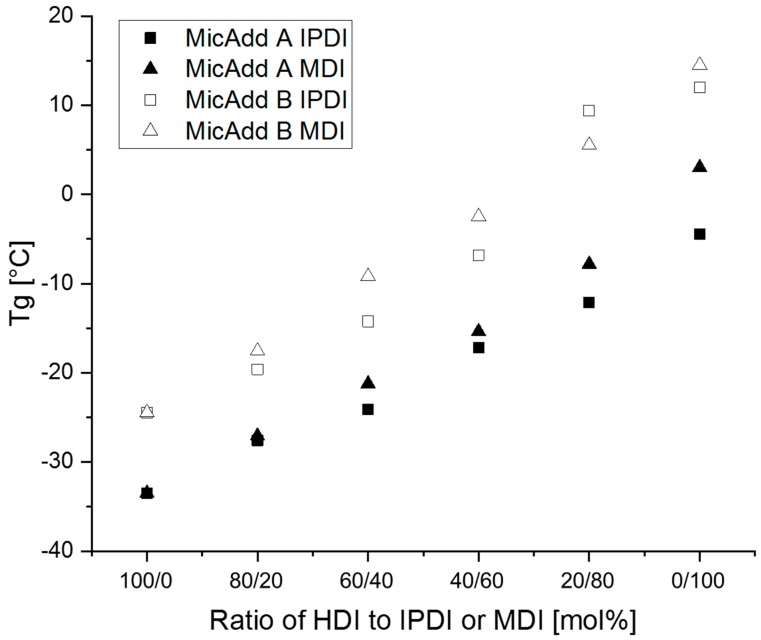
Change of T_g_ by altering ratios of diisocyanates in the reaction mixture.

The glass transition temperatures T_g_ were determined via DSC measurement with a heating rate of 10 K/min. Raising the amount of the IPDI/MDI showed a linear increase in T_g_ (Figure 3). Each series of polyurethanes started with a T_g_ of −33.5 °C for PU01A and −24.5 °C for PU01B. The polyurethane PU06B ended with a T_g_ of −4.4 °C. The T_g_ for PU11A was determined to be 3.0 °C. Polyurethanes containing MicAdd B (PU01B–11B) showed the same linear development ending with T_g_ at 12.0 °C for PU06B and 14.5 °C for PU11B.

The difference in carbon chain length effects the polymers glass transition temperatures as well as the viscosities. Polyurethanes containing MicAdd A (PU01A–11A) showed lower overall T_g_ values than MicAdd B-containing polyurethanes (PU01B–11B). In comparison, the T_g_ values of the MicAdd A series were 6–8 °C lower on average than those of the MicAdd B series.

### 3.3. Diels–Alder Reaction of N-Alkylated Maleimides with Linear PUs

**Scheme 3:**
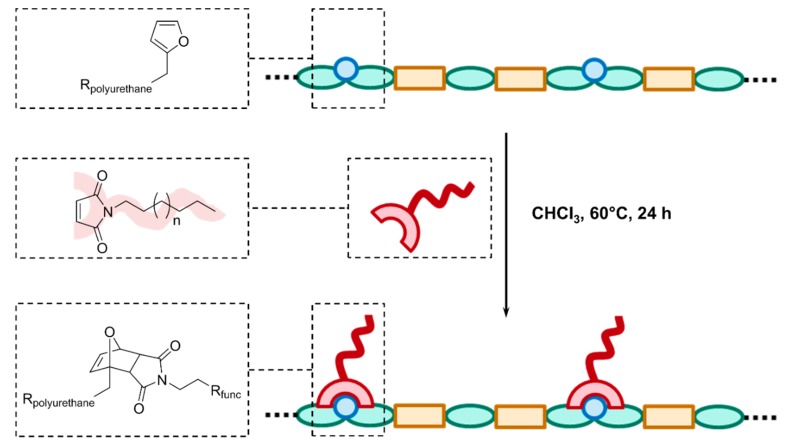
Diels–Alder reaction of the linear polyurethane with N-alkylated maleimides.

PU06A, PU11A, PU06B, and PU11B were selected for functionalization experiments. The functionalization was realized via Diels–Alder addition. The process is shown in Scheme 3. The polyurethanes and fatty maleimides were solved in chloroform to give a 40 wt% solution. N-alkyl maleimides were applied at a 0.8 ratio in relation to furan groups. The N-alkyl maleimides were obtained from maleic anhydride and fatty amines [18]. The reaction was carried out at 60 °C to avoid a retro Diels-Alder (DA) reaction, which started to occur at temperatures >80 °C.

**Figure 4:**
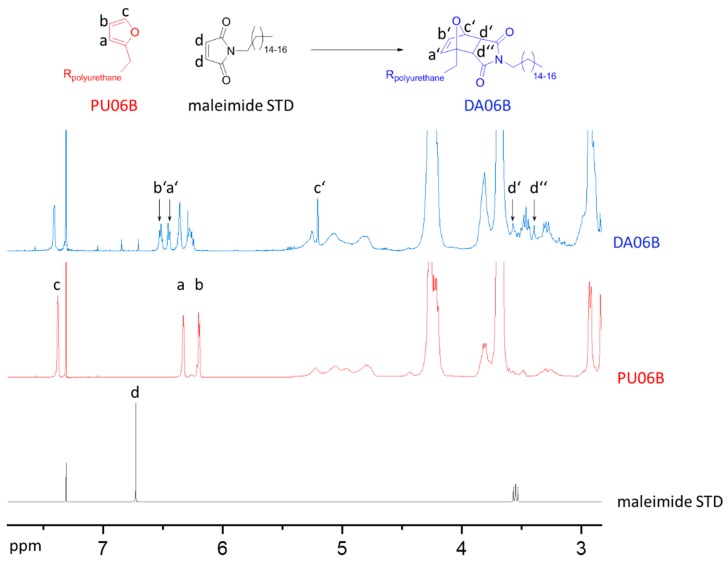
Section of changed ^1^H-NMR signals through the Diels–Alder reaction of linear polyurethane with maleimide.

Covalent bonding of the N-alkylated maleimides to the polyurethane was examined by ^1^H-NMR. The ^1^H-NMR spectra of the N-alkylated maleimide, the linear polyurethane, and the functionalized polyurethane are shown in Figure 4. Full spectra can be found in the Supplementary Information (Figures S1–S3). The maleimide double bond protons were assigned to the signal at 6.71 ppm (d). Corresponding furan signals of the polyurethane were determined at shifts of 7.38 ppm for H1 (c), 6.33 ppm for H2 (a), and 6.20 ppm for H3 (b). Change of the molecular structure was observed by several changes of peaks in ^1^H-NMR spectra of the product. The furan determined signals decreased but were still present. The same observations were made for the maleimide double bond protons. Beside the maleimide and the furan signals, new signals were found in the product spectra at shifts of 6.51 ppm (b’), 6.45 ppm (a’), 5.20 ppm (c’), 3.57 ppm (d’), and 3.39 ppm (d’’). The protons of the new formed double bond of the six membered ring were allocated at 6.51 ppm (dd, 1 H, *J* = 6.1, 12.2 Hz) and 6.45 ppm (d, 1 H, *J* = 6.1 Hz). The proton at the oxygen bearing bridge position was designated to 5.20 ppm (s, 1 H). The peaks at 3.55–3.58 ppm (m, 1 H) and 3.38–3.40 ppm (m, 1 H) were designated to the covalent bonded maleimide ring structure. The decrease of the peaks for the furan moiety and the newly formed peaks are evidence of a successful Diels–Alder addition of furan and maleimide. Despite having applied an excess of furan groups related to maleimides, we still found the double bond signal at 6.71 ppm, which belongs to non-converted maleimide. To determine the conversion towards the cycloaddition product, the integration of corresponding furan and maleimide peaks (c and d) was carried out. Integration showed a conversion of 97% of the maleimides.

By bonding hydrophobic groups to the polymeric backbone, the hydrophobicity of the polyurethane should increase. The hydrophobicity of the polyurethanes PU06A, PU11A, PU06B, and PU11A and of the functionalized polyurethanes DA06A, DA11A, DA06B, and DA11B were measured by the sessile drop method. Preparations of functionalized polyurethane samples for measurements were done by coating glass plates with a 250 μm layer of the functionalized polyurethanes. For comparison, unmodified polyurethanes were also prepared in a 40 wt% solution in chloroform and coated on the glass plates.

**Figure 5:**
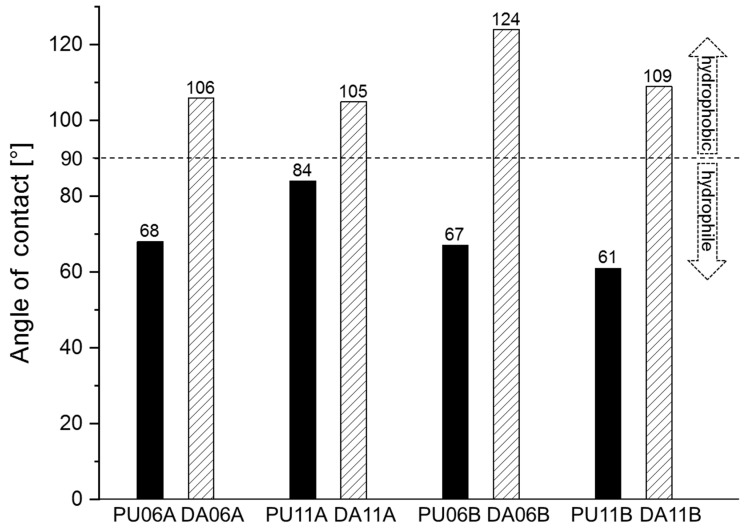
Contact angles of PUs and DA products.

Contact angles are summarized in Figure 5. Full data on contact angles with different solvents can be found in the Supplementary Information (Table S5). Contact angles between water and polyurethanes PU06A, PU11A, PU06B, and PU11B were lower than 90°. Materials with contact angles lower than 90° are defined as hydrophilic [40]. Contact angles of functionalized polyurethanes DA06A, DA11A, DA06B, and DA11B were above 90°. Materials with contact angles above 90° for water are determined to be hydrophobic. Therefore, the modified polyurethanes are considered to have hydrophobic surface properties.

**Figure 6:**
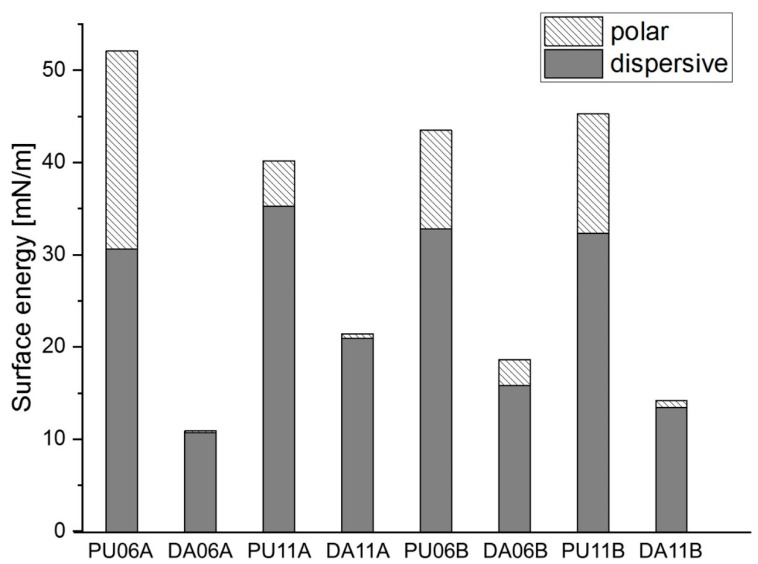
Surface energies of PUs and DA products.

Besides the contact angles of water, several other solvents (glycerine, ethylene glycol, formamide, bromonaphthalene, and diiodomethane) were measured. Data of the different solvent contact angles can be found in the Supplementary Information (Table S6). By processing the solvents’ surface interactions, surface energies of the polyurethanes can be determined. This process was done by utilizing the Young equation [41] shown in Equation (1), which describes the equilibrium of forces between liquid and solid phases. In combination with the Owens, Wendt, Rabel, and Kaelble Model (OWRK Model) [42,43,44], to involve known polar and disperse shares for the different tested liquids, the Young equation can be converted to a linear equation as shown in Equation 2. Via linear regression, the solid phase disperses and polar shares can be determined by the inclination and the y-axis intercept. Surface energies for the unmodified polyurethanes and for their Diels–Alder products are shown in Figure 6. Surface energies of the unmodified polyurethanes were in the range of 40–45 mN/m (PU06A, PU11A, PU06B, PU11B). The dispersive amount was about 30–35 mN/m. The polar share was lower overall with a value of 5–13 mN/m. By the hydrophobic functionalization of the polyurethanes, the surface energy decreased to 14–21 mN/m (DA06A, DA11A, DA06B, DA11B). The dispersive share dominated the surface properties after modification (13–20 mN/m), while polar shares were barely present (0.2–1 mN/m). In summary, the hydrophobic modification reduces the surface energy. In particular, the polar share decreased significantly. This was expected for a more hydrophobic surface due to its lower number of polar groups. Thus, Coulomb interactions are reduced and the polar share declines. The dispersive share is especially caused by van der Waals interactions.

(1) $$\sigma_{L}\cos\Theta_{C}=\;\sigma_{S}-\sigma_{SL},$$

(2) $$\frac{\sigma_{L}\left(1+\cos\Theta \right)}{2\sqrt{\sigma_{L}^{d}}}=\sqrt{\sigma_{S}^{p}}*\sqrt{\frac{\sigma_{L}^{p}}{\sigma_{L}^{d}}}+\sqrt{\sigma_{S}^{d}}.$$

## 4. Conclusions

In conclusion, we developed a method for the synthesis of functionalizable polyurethanes. The synthetic approach allows for a simple introduction of different functionalities to the polyurethane in a post-polymerization step.

The synthesis of linear furfurylated polyurethanes requires difunctional furan-containing building blocks. We synthesized a furan-containing diol utilizing furfurylamine and a hydroxyalkyl acrylate in a Michael addition reaction. These furan-containing diols were used for the synthesis of linear PUs in combination with different isocyanates and PEG 400. Different compositions of diisocyanates HDI, IPDI, and MDI as well as the Michael addition products were used to tune the physical properties. Viscosities and T_g_ values of the polyurethanes were measured to determine the influence of the monomers.

The functionalization was realized in a post-polymerization step via Diels–Alder addition. The total free surface energy decreased after modification. Moreover, the disperse and polar shares changed as well. The ratio of polar to dispersed shares was reduced significantly. Dispersed forces dominated the surface properties after Diels–Alder addition, which indicates the hydrophobicity of the surface.

In summary, this method allows for a simple introduction of different functionalities to furan-containing polyurethanes and could be used for highly customized products. Instead of a hydrophobic alkyl chain, the maleimides can also contain other functional groups with hydrophilic or antimicrobial characteristics. Via this method, several functional groups can be added to the polymer backbone. Thus, the synthetic approach described in this study allows for a simple introduction of different functionalities to a certain polymer in a post-polymerization step, imbuing the polymer with hydrophobic, hydrophilic, or antimicrobiological properties.

## Figures and Tables

**Table 1:** Isocyanate composition of linear polyurethanes.

Samples		Ratio of Isocyanates (mol%)			Amount of Furan Groups (mmol/g)	
with MicAdd A	with MicAdd B	HDI	IPDI	MDI	PUxxA	PUxxB
PU01A	PU01B	100	-	-	0.894	0.942
PU02A	PU02B	80	20	-	0.875	0.924
PU03A	PU03B	60	40	-	0.858	0.905
PU04A	PU04B	40	60	-	0.842	0.887
PU05A	PU05B	20	80	-	0.828	0.867
PU06A	PU06B	-	100	-	0.815	0.854
PU07A	PU07B	80	-	20	0.868	0.913
PU08A	PU08B	60	-	40	0.845	0.887
PU09A	PU09B	40	-	60	0.822	0.861
PU10A	PU10B	20	-	80	0.801	0.839
PU11A	PU11B	-	-	100	0.780	0.816

## Supplementary Materials

The following are available online at https://www.mdpi.com/2073-4360/11/8/1274/s1: Table S1. Determined compositions of reaction mixtures in experiments on synthesis of linear polyurethanes; Table S2. Compositions of reaction mixtures in experiments on the synthesis of linear polyurethanes; Table S3. Compositions of the functionalization of linear polyurethanes; Table S4. Measured viscosities and T_g_ values of polyurethanes; Table S5. Measured contact angles of different liquids for polyurethanes and Diels–Alder functionalized coatings; Table S6. Determined free surface energies for polyurethanes and Diels–Alder functionalized coatings; Figure S1. ^1^H-NMR of maleimide STD; Figure S2. ^1^H-NMR of PU06B; Figure S3. ^1^H-NMR of DA06B.
