# Supplementary material for: Hydrophobilization of Furan-Containing Polyurethanes via Diels–Alder Reaction with Fatty Maleimides
Source: Polymers (Basel). 2019 Jul 31;11(8):1274. doi: 10.3390/polym11081274 (PMC6723512; doi:10.3390/polym11081274)
Supplement: Supplementary file 1 [file polymers-11-01274-s001.pdf]

Hydrophobilization of furan containing polyurethanes via Diels-Alder reaction with fatty maleimides

Philipp Schmidt [1], Steven Eschig [2,*]

[1] Fraunhofer Institute for Wood Research, Wilhelm-Klauditz-Institut WKI; philipp.schmidt@wki.fraunhofer.de
[2] Fraunhofer Institute for Wood Research, Wilhelm-Klauditz-Institut WKI; steven.eschig@wki.fraunhofer.de
* Correspondence: steven.eschig@wki.fraunhofer.de; Tel.: +49 531 2155433

1. Materials and Methods

1.1 Instruments

^{1}H-NMR and ^{13}C-NMR spectra were recorded on Bruker AV III-400 (400.1 MHz for ^{1}H, 100.6 MHz for ^{13}C, T = 296 K), Bruker DRX-400 (400.1 MHz for ^{1}H, 100.6 MHz for ^{13}C, T = 300 K) and Bruker AV II-600 (600.1 MHz for ^{1}H, 150.9 MHz for ^{13}C, T = 295 K) using $CDCl_3$ or THF-d_8 as solvent. Chemical shifts are expressed in ppm with TMS (0.00 ppm) as an internal standard for ^{1}H. ^{13}C-NMR spectra are referenced to $CDCl_3$ (77.0 ppm) or THF-d_8 (67.2 ppm and 15.3 ppm). IR spectra were measured with Nicolet iS5 iD7 ATR (ThermoFisher Scientific). Mass spectra (ESI-MS/EI-MS) were performed on spectrometers Finnigan MAT 95 XL (Thermofinnigan MAT) and LTQ Orbitrap Velos (ThermoFisher Scientific). Viscosities were measured on Bohlin CVO 100 Rheometer at 90 °C with constant shearing stress of 100 Pa. Contact angle measurement was achieved by sessile drop method on DataPhysics Instruments SCA20. T_g's were measured on Mettler Toledo DSC 3+ in the range of -70 to 180 °C under nitrogen atmosphere. The heating rate was kept at 10 K/min. DSC data were processed by Mettler STARe software and exploited by ASTM E1356 standards.

1.2 Chemicals

Solvents and reagents were obtained from Carl Roth (Polyethylene glycol 400 (PEG400)), Alfa Aesar (2-hydroxyethyl acrylate (HEA), furfurylamine, 1,1'-methylenebis(4-isocyanatobenzene) (MDI)) and VWR International (chloroform). 4-Hydroxybutyl acrylate was provided by BASF and 5-isocyanato-1-(isocyanatomethyl)-1,3,3-trimethylcyclohexane (IPDI) by Evonik Industries. 1,6-Diisocyanatohexane (HDI) was obtained by Perstop AB. Borchikat® 0244 was provided by OMG Borchers GmbH. Maleimide STD was achieved by reaction of maleic anhydride (Alfa Aesar) with a mixture of fatty amines (Rofamin STD) provided by Ecogreen Oleochemicals. All chemicals had synthesis quality and were used without further purification.

1.3 Procedure for Synthesis of Michael Products

1.3.1 MicAdd A

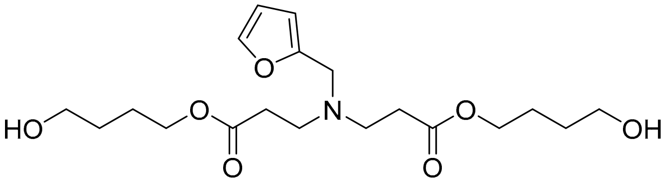

A 500 mL round bottom flask was charged with 4-Hydroxybutyl acrylate (144.23. g, 1.00 mol, 2 eq.) and cooled in a bath of iced water. A dropping funnel with pressure compensation was filled with furfurylamine (48.60 g, 0.50 mol, 1 eq.) and fitted to the round bottom flask. Under constant ice bath cooling and vigorous stirring furfurylamine was added dropwise over 1 h to the hydroxyalkyl acrylate. The solution was stirred for additional 3 h under cooling in an ice bath. Afterwards the reaction mixture was stirred for 6 days at room temperature (rt). Progress of the reaction was tracked by TLC. After

6 days the product was obtained as yellow oil in quantitative yields. The product was used without further purification in the next step.

.

TLC [EA 100 %]: R_f = 0.42.
^{1}H-NMR (400 MHz, $CDCl_3$) δ = 7.41 (d, 1 H, J = 1.5 Hz, OC*H*), 6.36 (dd, 1 H, J = 1.9, 3.2 Hz, OCHC*H*), 6.23 (d, 1 H, J = 4.0 Hz, OCHCHC*H*), 4.16 (t, 4 H, J = 6.2 Hz, (CO)OCH_2), 3.74 (s, 2 H, OCCH_2), 3.74 (t, 4 H, J = 6.4 Hz, HOCH_2), 2.84 (t, 4 H, J = 7.0 Hz, COCH_2CH_2),2.52 (t, 4 H, J = 7.0 Hz, (CO)CH_2), 1.80-1.73 (m, 4 H, (CO)OCH_2CH_2), 1.71-1.64 (m, 4 H, HOCH_2CH_2).
^{13}C-NMR (100 MHz, $CDCl_3$) δ = 172.6 (2 C, (CO)O), 151.8 (OC), 142.0 (OCH), 110.8 (OCCH), 108.7 (OCHCH), 64.3 (2 C, (CO)OCH_2), 62.3 (2 C, HOCH_2), 49.4 (OCCH_2), 49.1 (2 C, (CO)CH_2CH_2), 33.0 (2 C, (CO)CH_2), 29.1 (2 C, (CO)OCH_2CH_2), 25.1 (2 C, HOCH_2CH_2).
IR (diamond-ATR): $\tilde{\nu}$ = 3396 (w), 2944 (w), 1725(s), 1503 (w), 1392 (w), 1259 (m), 1172 (s), 1147 (m), 1113 (w), 1041 (m), 1011 (m), 942 (m), 884 (w), 810 (w), 735 (m), 600 (m), 446 (w).
HRESIMS: *m*/*z* $[M + Na]^+$ calcd. for $C_{19}H_{31}NO_7Na$: 408.19927; found: 408.19960.

1.3.2 MicAdd B

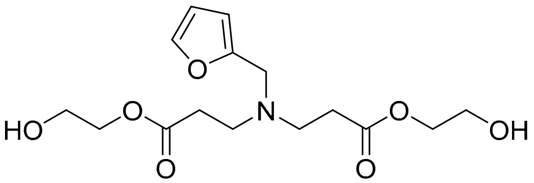

A 2000 mL round bottom flask was charged with 2-Hydroxyethyl acrylate (809.36 g, 6.974 mol, 2 eq.) and cooled in a bath of iced water. A dropping funnel with pressure compensation was filled with furfurylamine (338.71 g, 3.487 mol, 1 eq.) and fitted to the round bottom flask. Under constant ice bath cooling and vigorous stirring furfurylamine was added dropwise over 1 h to the hydroxyalkyl acrylate. The solution was stirred for additional 3 h under cooling in an ice bath. Afterwards the reaction mixture was stirred for 6 days at room temperature (rt). Progress of the reaction was tracked by TLC. After 6 days the product was obtained as yellow oil in quantitative yields. The product was used without further purification in the next step.

TLC [$CHCl_3$/MeOH (9:1)]: R_f = 0.6.
^{1}H-NMR (400 MHz, $CDCl_3$) δ = 7.42 (dd, 1 H, *J* = 0.8, 1.8 Hz, OC*H*), 6.37 (dd, 1 H, *J* = 2, 3.2 Hz, OCHC*H*), 6.26 (d, 1 H, *J* = 3.2 Hz, OCC*H*), 4.29-4.28 (m, 4 H, (CO)OCH_2), 3.84-3.82 (m, 4 H, HOCH_2), 3.77 (s, 2 H, OCCH_2), 2.85 (t, 4 H, *J* = 6.8 Hz, (CO)CH_2CH_2), 2.58 (t, 4 H, *J* = 6.8 Hz, (CO)CH_2).
^{13}C-NMR (100 MHz, $CDCl_3$) δ = 172.6 ((CO)O), 150.9 (OC), 142.3 (OCH), 110.2 (OCCH), 109.4 (OCHCH), 66.0 (2 C, (CO)OCH_2), 60.8 (2 C, HOCH_2), 49.4 (OCCH_2), 49.3 (2 C, (CO)CH_2CH_2), 33.0 (2 C, (CO)CH_2).
IR (diamond-ATR): $\tilde{\nu}$ = 3392 (w), 2950 (w), 1726 (s), 1502 (w), 1454 (w), 1386 (w), 1172 (s), 1147 (m), 1074 (s), 1043 (m), 1012 (s), 957 (w), 916 (w), 883 (m), 813 (m), 737 (m), 600 (m), 504 (m).
HRESIMS: *m*/*z* $[M + Na]^+$ calcd. for $C_{15}H_{23}NO_7Na$: 352.13667; found: 352.13696.

1.4 General Procedure for synthesis of linear Polyurethanes

A 100 mL three necked round bottom flask was charged with PEG 400, the furfuryl-containing Michael-Addition product and the diisocyanates (HDI, MDI, IPDI). The sum of all initial weights was 20 g. The molar ratio of PEG400 and Michael-adducts was defined as 1:1. The NCO/OH value was set to 1:1. The molar diisocyanate ratios of the different batches are summarized in Table 1. The reaction mixture was solved in dry acetone (12 ml) and diluted to give a 60 wt% solution. The solution was heated up to 60 °C while stirring. Depending on viscosity of the mixture additional acetone was added. At 60 °C 0.06 m% Borchikat® 0244 was added to the mixture and stirred for 4 h. After 4 h reaction progress was

checked by IR. With depletion of isocyanates the reaction was finished giving the polyurethanes as yellow resins. The products were used without further purification.

Table S1 Determined compositions of reaction mixtures in experiments on synthesis of linear polyurethanes.

Experiment		Diol [%]			Diisocyantes [%]		Borchikat 0244 [m%]
	PEG 400	MicAdd A	MicAdd B	HDI	MDI	IPDI	
PU01A	50	50	0	100	0	0	0.06
PU02A	50	50	0	80	0	20	0.06
PU03A	50	50	0	60	0	40	0.06
PU04A	50	50	0	40	0	60	0.06
PU05A	50	50	0	20	0	80	0.06
PU06A	50	50	0	0	0	100	0.06
PU07A	50	50	0	80	20	0	0.06
PU08A	50	50	0	60	40	0	0.06
PU09A	50	50	0	40	60	0	0.06
PU10A	50	50	0	20	80	0	0.06
PU11A	50	50	0	0	100	0	0.06
PU01B	50	0	50	100	0	0	0.06
PU02B	50	0	50	80	0	20	0.06
PU03B	50	0	50	60	0	40	0.06
PU04B	50	0	50	40	0	60	0.06
PU05B	50	0	50	20	0	80	0.06
PU06B	50	0	50	0	0	100	0.06
PU07B	50	0	50	80	20		0.06
PU08B	50	0	50	60	40		0.06
PU09B	50	0	50	40	60		0.06
PU10B	50	0	50	20	80		0.06
PU11B	50	0	50	0	100	0	0.06

Table S2 Compositions of reaction mixtures in experiments on synthesis of linear polyurethanes.

Experiment	PEG 400 [g]	MicAdd A [g]	MicAdd B [g]	HDI [g]	MDI [g]	IPDI [g]	Borchikat 0244 [mg]
PU01A	7.14	6.89	0	5.99	0	0	0.011
PU02A	7.01	6.74	0	4.73	0	1.57	0.013
PU03A	6.87	6.61	0	3.48	0	3.06	0.014
PU04A	6.74	6.49	0	2.28	0	4.51	0.012
PU05A	6.64	6.38	0	1.12	0	5.89	0.014
PU06A	6.51	6.28	0	0	0	7.25	0.012
PU07A	6.93	6.69	0	4.68	1.73	0	0.012
PU08A	6.76	6.51	0	3.42	3.37	0	0.013
PU09A	6.56	6.33	0	2.23	4.92	0	0.014
PU10A	6.39	6.17	0	1.09	6.41	0	0.013
PU11A	6.22	6.01		0	7.81	0	0.015
PU01B	7.53	0	6.20	6.32	0	1.64	0.014
PU02B	7.37	0	6.08	4.96	0	3.23	0.014

PU03B	7.23	0	5.96	3.64	0	4.73	0.012
PU04B	7.09	0	5.84	2.40	0	6.96	0.013
PU05B	6.94	0	5.71	1.18	0	7.60	0.015
PU06B	6.84	0	5.62	0	0	0	0.015
PU07B	7.51	0	6.01	4.92	1.84	0	0.012
PU08B	7.09	0	5.84	3.58	3.56	0	0.013
PU09B	6.89	0	5.67	2.33	5.16	0	0.012
PU10B	6.70	0	5.52	1.12	6.71	0	0.014
PU11B	6.53	0	5.37	0	8.15	0	0.014

1.5 General Procedure for Diels-Alder-Reaction of linear Polyurethanes

A 50 mL round bottom flask was charged with polyurethane (2-3 g) and 80 mol% of maleimide STD related to the quantity of furan groups in the polyurethane. The reactants were solved in 3-4 ml chloroform and diluted to a 40 wt% solution. The solution was refluxed under constant stirring for 16 h. After end of reaction a plain glass plate was coated with a 250 µm layer of the solution. Then the product was dried for 2-3 days at rt. After drying the product appeared as light yellow resin.

Table S3 Compositions of functionalization of linear polyurethanes.

**Experiment**		**Composition**		**Temp.**	**m(PU)**	**m(Mal)**	**Amount**	**Maleimide**
	**PU**	**Maleimide**	**Solvent**	**[°C]**	**[g]**	**[g]**	**FU [mmol/g]**	**: Furan**
DA01A	1A	Mal STD	$CHCl_3$	60	3.1575	0.7857	0.892	0.8
DA06A	6A	Mal STD	$CHCl_3$	60	2.4079	0.5462	0.813	0.8
DA11A	11A	Mal STD	$CHCl_3$	60	2.3104	0.5143	0.798	0.8
DA01B	01B	Mal STD	$CHCl_3$	60	2.5879	0.5787	0.939	0.8
DA06B	06B	Mal STD	$CHCl_3$	60	2.5282	0.5140	0.853	0.8
DA11B	11B	Mal STD	$CHCl_3$	60	2.5628	0.4971	0.814	0.8

2 Viscosities, T_g and ^{1}H-NMR data.

2.1 Viscosities and T_g

Table S4 Measured viscosities and T_g for polyurethanes

**Experiment**	**Viscosity [Pas]**	**T_g [°C]**	**Amount of contained Furan groups [mmol/g]**
PU01A	7.73	-33.5	0.894
PU02A	6.84	-27.6	0.875
PU03A	12.40	-24.1	0.858
PU04A	23.38	-17.2	0.842
PU05A	98.33	-12.1	0.828
PU06A	341.69	-4.4	0.815
PU07A	18.15	-27.1	0.868
PU08A	88.75	-21.2	0.845
PU09A	142.53	-15.4	0.822
PU10A	435.57	-7.8	0.801
PU11A	1308.04	3.0	0.780
PU01B	78.31	-24.5	0.942
PU02B	134.24	-19.6	0.924
PU03B	180.61	-14.3	0.905

PU04B	405.06	-6.8	0.887
PU05B	/	10.4	0.867
PU06B	11504.80	12.0	0.854
PU07B	686.96	-17.6	0.913
PU08B	1233.67	-9.2	0.887
PU09B	1916.14	-2.5	0.861
PU10B	2899.97	5.5	0.839
PU11B	5777.81	14.5	0.816

2.2 Contact angle measurement

Table S5 Measured contact angles of different liquids for polyurethane and Diels-Alder functionalized coatings.

**Liquid**	**Contact angle Θ_c [°]**							
	**PU06A**	**PU11A**	**PU06B**	**PU11B**	**DA06A**	**DA11A**	**DA06B**	**DA11B**
Water	68±2	84±7	67±3	61±2	106±1	105±1	124±14	109±7
Glycerine	67±1	63±1	63±1	60±4	97±1	96±1	106±7	97±2
Ethylene glycol	43±1	55±5	23±4	43±3	82±1	83±1	118±5	95±14
Formamide	38±1	44±13	41±2	21±5	93±1	92±1	116±1	106±11
Bromonapthalene	16±3	23±7	17±3	19±2	62±1	58±10	54±24	84±20
Diiodomethane	47±8	48±	44±6	35±2	72±2	71±3	77±7	81±14

Table S6 Determined free surface energys for polyurethane and Diels-Alder functionalized coatings.

	**PU06A**	**PU11A**	**PU06B**	**PU11B**	**DA06A**	**DA11A**	**DA06B**	**DA11B**
Surface energy [Nm/m]	41.4±1.2	40.2±1.3	43.5±1.2	45.3±1.6	21.7±0.7	21.4±3.1	18.6±14.1	14.2±2.6
Disperse	30.6±3.4	35.3±5.6	32.8±0.7	32.3±0.9	21.5±0.8	20.9±3.5	15.8±11.6	13.4±2.5
Polar	10.7±2.3	4.9±4.6	10.7±1.1	13.0±2.0	0.2±0.1	0.5±0.4	2.8±2.5	0.8±0.3

2.3 1^H-NMR Spectra

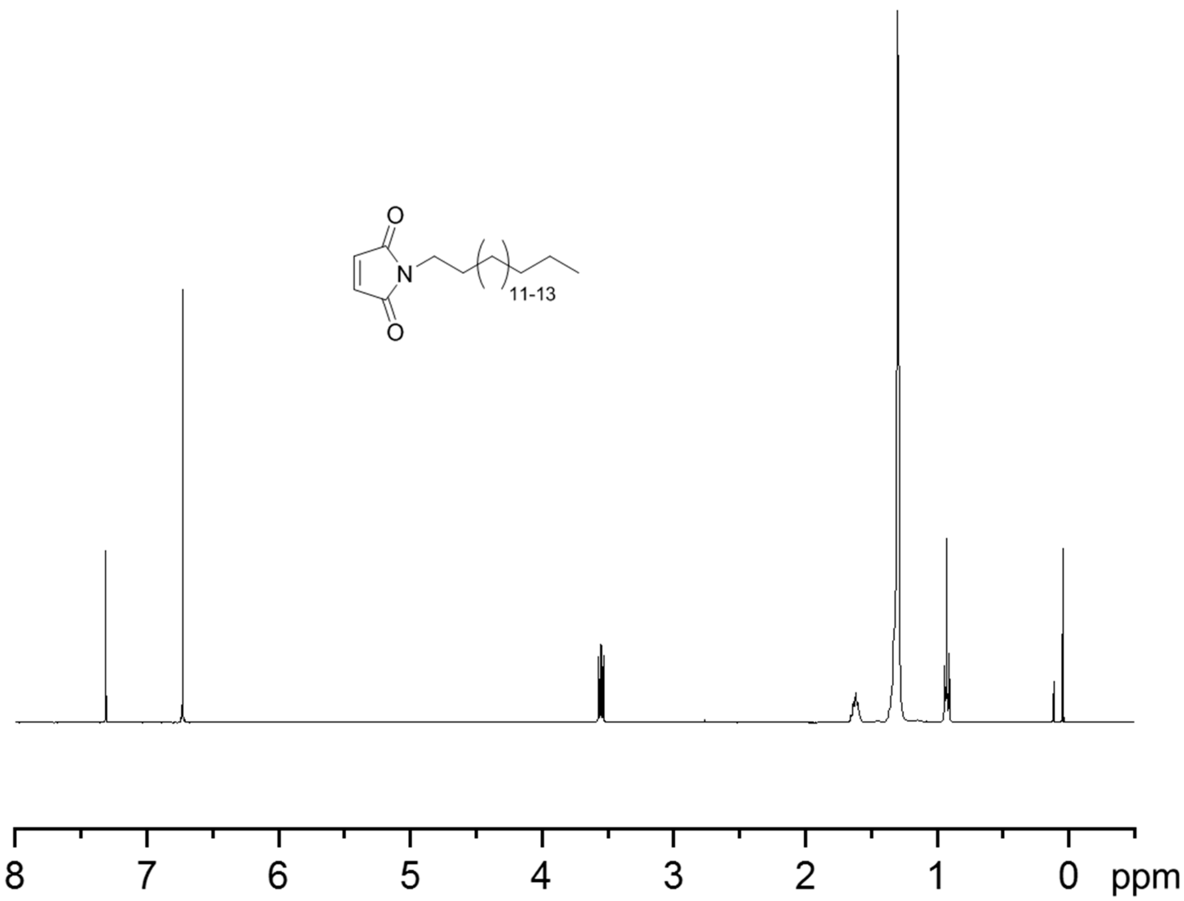

Figure S1 ^{1}H-NMR of maleimide STD; Solvent: $CDCl_3$.

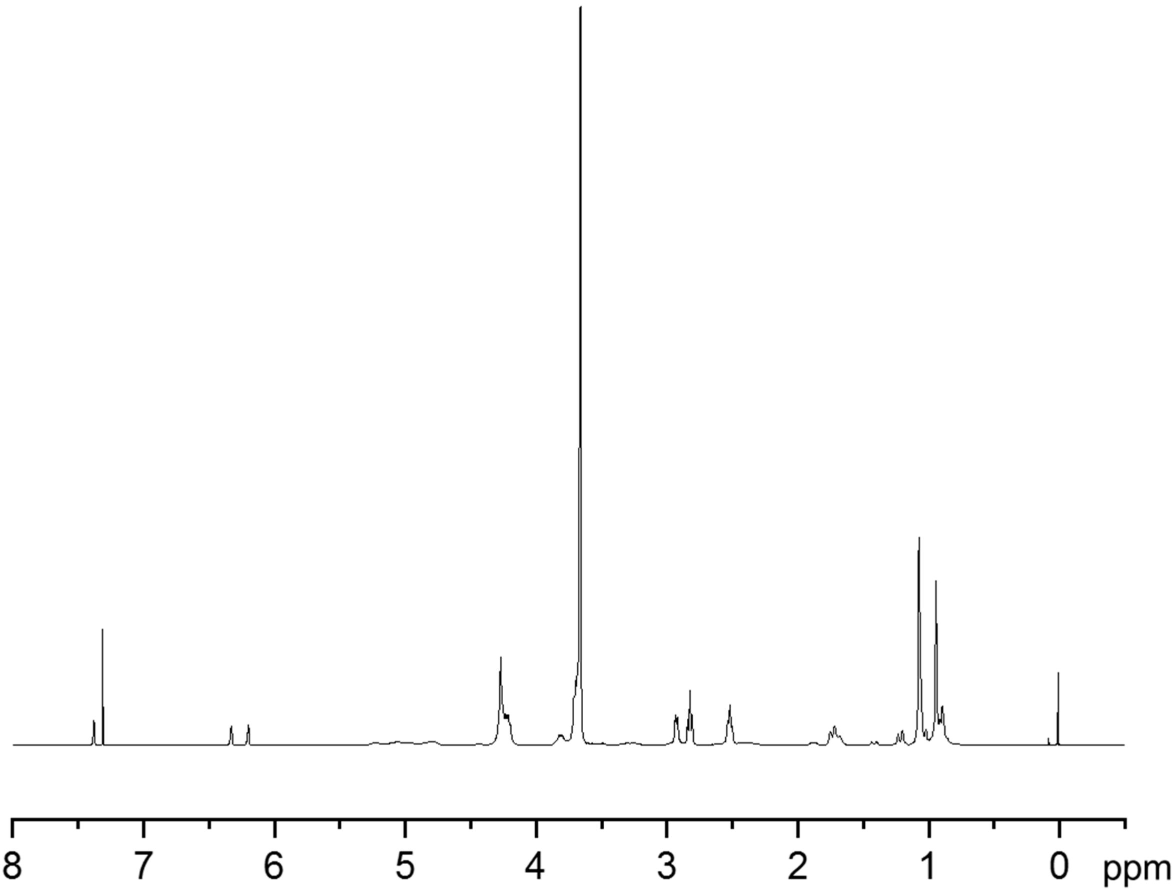

Figure S2 ^{1}H-NMR of PU06B; Solvent: $CDCl_3$.

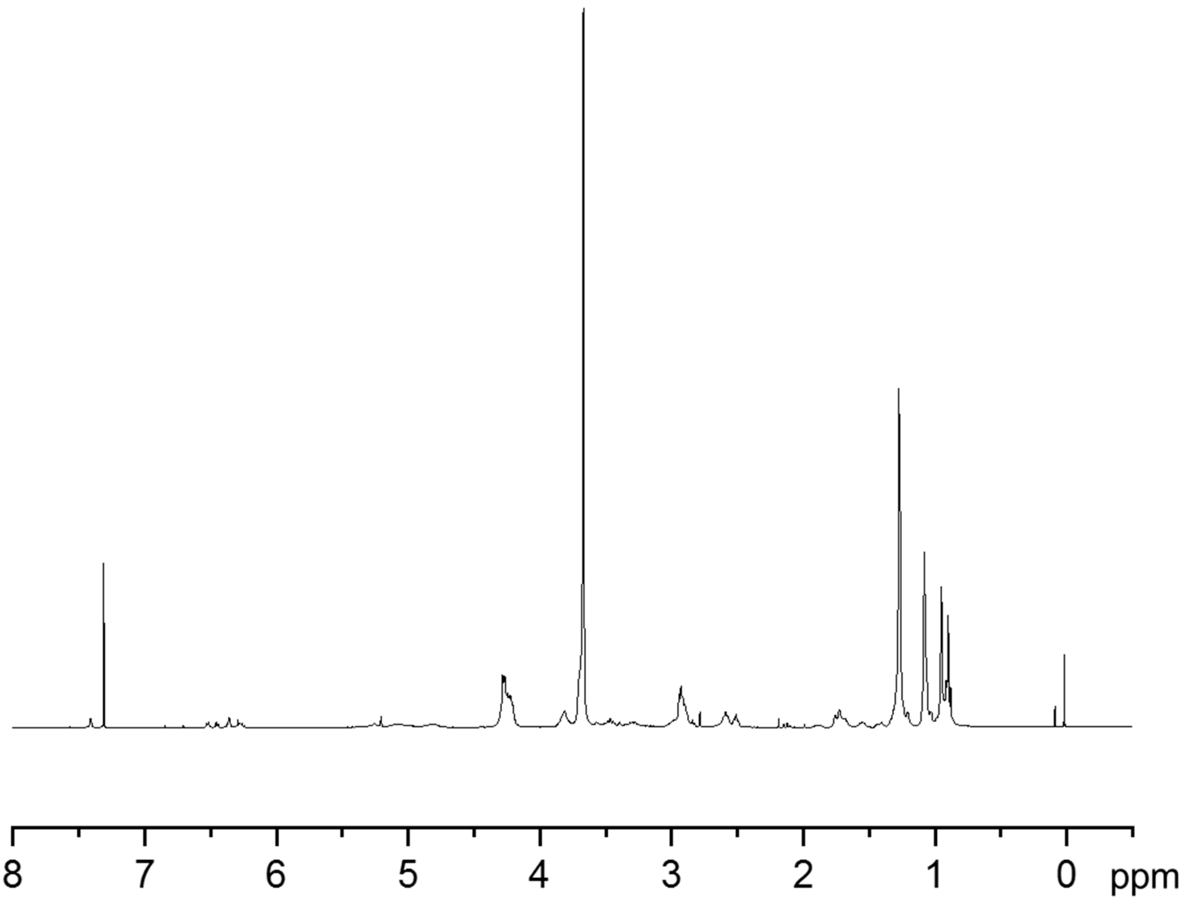

Figure S3 ^{1}H-NMR of DA06B; Solvent: $CDCl_3$.
